# Protocol for interpretable and context-specific single-cell-informed deconvolution of bulk RNA-seq data

**DOI:** 10.1016/j.xpro.2025.103670

**Published:** 2025-03-04

**Authors:** Daniele Malpetti, Francesca Mangili, Marco Bolis, Anna Rinaldi, David Legouis, Lorenzo Ruinelli, Pietro Cippà, Laura Azzimonti

**Affiliations:** 1 Istituto Dalle Molle di Studi sull'Intelligenza Artificiale (IDSIA), SUPSI, 6900 Lugano, Switzerland; 2 Institute of Oncology Research, Università della Svizzera Italiana, Bellinzona, Switzerland; 3 Laboratory of Computational Oncology, Department of Oncology, Istituto di Ricerche Farmacologiche Mario Negri, Milano, Italy; 4 Laboratories for Translational Research, Ente Ospedaliero Cantonale, Bellinzona, Switzerland; 5 Division of Nephrology, Department of Medicine, Ente Ospedaliero Cantonale, 6900 Lugano, Switzerland; 6 Division of Intensive Care, Department of Acute Medicine, University Hospital of Geneva, 1205 Geneva, Switzerland; 7 Laboratory of Nephrology, Department of Physiology and Cell Metabolism, University of Geneva, 1205 Geneva, Switzerland; 8 Ente Ospedaliero Cantonale, 6900 Lugano, Switzerland; 9 Faculty of Biomedical Sciences, Università della Svizzera Italiana, 6900 Lugano, Switzerland

**Keywords:** Bioinformatics, Single Cell, RNA-seq

## Abstract

Single-cell sequencing provides rich information; however, its clinical use is limited due to high costs and complex data output. Here, we present a protocol for extracting single-cell-related information from bulk RNA-sequencing (RNA-seq) data using the pathway-level information extractor (PLIER) algorithm. We describe the steps for extracting single-cell signatures from literature, training a PLIER model based on single-cell signatures (named CLIER), and applying it to a new dataset. This produces latent variables that are interpretable in the context of specific single-cell biology.

For complete details on the use and execution of this protocol, please refer to Legouis et al.,^1^ where this approach is used within the renal context.

## Highlights

• Guidelines for constructing a single-cell signature atlas
• Procedure for learning a single-cell-informed deconvolution specific to a given context
• Steps for using the learned deconvolution to transform a new dataset
• Instructions for possible analyses that can be performed on the transformed dataset

## Abstract (editor highlights)

Publisher’s note: Undertaking any experimental protocol requires adherence to local institutional guidelines for laboratory safety and ethics.

## Graphical abstract

**Figure:**
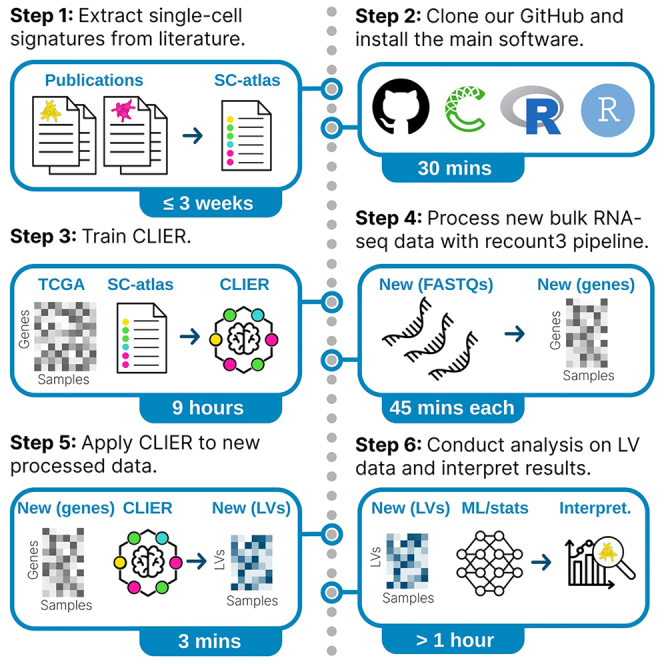

## Before you begin

A challenge in bulk RNA-seq analysis is extracting biological insights, such as variations in cell type proportions. In contrast, single-cell analysis allows for new biological discoveries but has limited applicability in large-scale studies.

In this protocol we show how to apply the pathway-level information extractor (PLIER), a generally applicable unsupervised transfer learning method developed by Mao et al.,^2^ to extract biological information related to cell types and states from bulk RNA-seq. PLIER leverages a bulk RNA-seq gene expression training dataset and an atlas of gene sets (such as pathways or single-cell signatures) to learn a transformation capable of producing a low-dimensional representation of high-dimensional bulk RNA-seq data. This transformation can then be applied to other datasets, to reduce their dimensionality to a relatively small set of latent variables (LVs), some of which are interpretable as they are associated to gene sets in the input atlas. These LVs can be handled as new features and correlated with clinical data of interest to obtain interpretable biological models.

From a technical perspective, PLIER decomposes the gene expression matrix into the approximating matrix product C U B. Each column of Y represents a sample, while the rows correspond to genes. C is a matrix containing gene sets (specifically, single-cell signatures in this protocol) as columns. U is a matrix with as many columns as the number of latent variables (LVs) and as many rows as the number of signatures, which defines the associations between LVs and signatures and is key for interpreting the LV biological meaning. B is a matrix whose columns are the samples projected in the LVs space. Importantly, B has a lower dimensionality compared to Y. The transformation is learned by training on Y in a way that minimizes the reconstruction error of Y while encouraging Z to align as much as possible with the product C U. Although there are no guarantees for convergence during training, it typically occurs in a wide range of scenarios. The choice of the signatures, while important for the interpretability of the model, does not impact convergence. After training, the transformation can be applied to a new gene expression dataset Y′, thus factorizing it as C U B’. For more details on the algorithm, its mathematical formulation, and its performance in different controlled settings, we refer readers to the original works by Mao et al.^2^^,^^3^

We demonstrate the protocol using the TCGA dataset for training PLIER. Given its large size and the variety of samples it contains, TCGA is a reasonable training dataset in a wide range of situations. However, it is not the only option. Other large-scale datasets, e.g., those included in the recount project,^4^ can be used instead of or in addition to the TCGA for training PLIER. As a gene sets atlas, we use the atlas of cell types and states of human kidney disease developed in Legouis et al.^1^ to tailor the PLIER for the interpretation of kidney data. This is an atlas specific to a given domain, as the main aim of the protocol id to generate a context-specific model.

We will call a model trained with single-cell signatures CLIER, where the “C” highlights the focus on cell signatures rather than pathways, and K-CLIER the specific CLIER trained with kidney signatures.

The transformation of a new dataset using the trained model includes the processing of the FASTQ reads with the recount3 pipeline to get TPM data consistent with the training data, and the application of learned transformation to the resulting dataset. The K-CLIER is applied, for demonstration purposes, to the GSE142025/SRP23754 dataset, which we also used in the main manuscript.^1^ We will refer to that dataset as the DKD dataset, in the same way as in the main manuscript.

### Institutional permissions

The use of previously published single-cell signatures or bulk RNA-seq data does not require approval. Any experiment carried out to extract new single-cell signatures needs to be approved by the local ethical committee. Similarly, the use of any bulk RNA-seq patient data, as well as clinical data, requires approval from an ethical committee.

### Extracting single-cell signatures from literature

**Timing: approx. 3 weeks to include 725 signatures from 15 publications (time might be shortened to 1 or 2 weeks if signature atlases are available)**

This major step details the procedures for extracting specific single-cell signatures in cases where a signature atlas tailored to the problem of interest is unavailable.

1. Conduct a comprehensive literature review to identify articles that feature single-cell signatures or atlases relevant to your domain of interest.

***Note:*** Apply well-defined criteria to select datasets, considering the species of interest and ensuring the inclusion of both negative and positive controls. For example, in our study, we examined human specimens derived from both non-pathological and diseased kidneys, with a focus on signatures related to inflammation and fibrosis.

2. Assess the selected articles to confirm they align with your research objectives and adequately address the relevant biological context.

***Note:*** Prioritize studies where identified cell types and states are validated using complementary techniques such as histological analysis, Real-Time PCR, Western Blotting of sorted cells, or spatial transcriptomics. These validation methods enhance the robustness and credibility of the gene signatures.

**CRITICAL:** Ensure the single-cell atlas includes signatures that comprehensively represent all aspects of the biology of interest. If key information is lacking, plan to conduct a new sequencing experiment to generate the required signatures. For more details, consult problem 1 in the troubleshooting section.

3. For each selected article, extract the single-cell signatures of interest if they are readily available.

***Note:*** If the signatures are not directly provided, reanalyze the data to derive them. For differential gene expression analysis, we encouraged methods that account for variation between biological replicates.^5^ In instances where the cell count is low, you may consider using methods that are not dependent on the number of cells to ensure reliable signature extraction.^6^ Top performing methods shared a common analytical property. These methods aggregate cells within a biological replicate.

4. Construct a matrix with genes on rows and the selected signatures on columns.

***Note:*** Mark each column with zeros and ones, where one indicates the inclusion of the gene in the signature. Store this matrix in an RDS file named atlas_matrix.rds.

5. Create an Excel file atlas_info.xlsx where to include essential signature details to facilitate further analysis and interpretation of the low-dimensional datasets that will be obtained through the transformation.

***Note:*** For example, for each signature, include reference, main associated cell type, and other useful categories relevant to your research focus.

### Cloning our GitHub repository and installing the main software

**Timing: 30 min**

This major step is dedicated to importing code and installing the main software to carry out the analysis.

6. Open your terminal and clone our GitHub repository using

> git clonehttps://github.com/IDSIA/CLIER

***Note:*** The repository https://github.com/IDSIA/CLIER contains all the necessary scripts to execute the protocol, apart from the preliminary genetic data processing step, as well as an example atlas of single-cell signatures (focused on kidney biology), “atlas.xsls,” which contains single-cell signatures and their descriptions, and an example dataset “DKD_clin.rds” which contains clinical information for the DKD dataset which is used as example throughout this protocol. Both are extracted from the main publication associated with this protocol.^1^

***Note:*** We do not provide raw FASTQ files in the repository because their size amounts to several gigabytes. Instead, we provide an already processed TPM version of the DKD dataset (“DKD_tpm.rds”). If needed, FASTQ files can be downloaded using the SRA-toolkit (https://github.com/ncbi/sra-tools) or sra-explorer (https://github.com/ewels/sra-explorer). The code to download the example files used in this protocol is provided in step 15.

***Note:*** Readers interested in executing specific steps of the pipeline that require files generated in previous steps, without running the entire pipeline, can download these files from the cloud storage platform Switchdrive (https://drive.switch.ch/index.php/s/OpvMh1vGRgRmKKf). The available intermediate files are: “tcga.rds”, containing a TPM version of TCGA; “associations.xlsx”, containing the associations of LVs signatures formatted as a table; “kclier.rds”, containing a trained KCLIER model; and “DKD_B.rds”, containing the LV expression of the DKD dataset (obtained through KCLIER).

***Note:*** Some instructions executed in the process print content on the screen. For the sake of simplicity, we include this content in the code snippets only when it is relevant for understanding the procedure.

7. If they are not installed, install R (https://www.r-project.org/) and if desired RStudio (https://www.rstudio.com/).
8. Install conda (https://conda.io/projects/conda/en/latest/user-guide/install/index.html).

## Key resources table

**Table:** 

REAGENT or RESOURCE	SOURCE	IDENTIFIER
**Deposited data**		

The Cancer Genome Atlas	The National Cancer Institute’s (NCI’s) Genomic Data Commons (GDC)	https://gdc.cancer.gov/access-data
DKD cohort (as an example dataset)	GEO	GEO: GSE142025

**Software and algorithms**		

R version 4	R Development Core Team, 2011	https://www.r-project.org/
conda	Anaconda, Inc.	https://conda.io/projects/conda/en/latest/user-guide/install/index.html
STAR 2.7.10a	Bioconda	Dobin et al.^7^
recount3 (version 1.14.0), recount (version 1.30.2)	Bioconductor	Wilks et al.^4^
BioMart (version 2.60.1)	Bioconductor	https://www.ensembl.org/info/data/biomart/index.html
PLIER (version 0.99.0)	https://github.com/wgmao/PLIER	Mao et al.^2^

**Other**		

Server or local station (see materials and equipment for details)	N/A	N/A

## Materials and equipment

The processing of genetic data was conducted on a server (2x 24 Core Intel CascadeLake 8260 Processor, 768 GB DDR4 2933MHz ECC Server Memory).

STAR is known for its speed and high accuracy in RNA-seq alignment but is also memory-intensive, especially for large genomes like human and mouse, as outlined in the STAR manual (https://github.com/alexdobin/STAR/blob/master/doc/STARmanual.pdf).

Users with limited computational resources, such as local stations, can refer to problem 2 of the troubleshooting section.

The training of PLIER and all subsequent analysis steps were also conducted on a server (2x 16 Core Intel XEON 6326 2.9 GHz Processor, 512 GB DDR4 3200 MHz ECC Server Memory). Although conducted on a server in our case, these analyses could also be performed on a personal computer without a significant impact on the time required for execution.

## Step-by-step method details

### Training PLIER algorithm

**Timing: approx. 9 h**

This major step performs the training phase of the PLIER algorithm. It takes as input the previously generated single-cell signatures atlas, downloads the TCGA dataset, trains a CLIER, and performs a few postprocessing steps.

***Note:*** The outputs include a TCGA dataset in TPM, a CLIER model (the K-CLIER in this case), an Excel file to guide the interpretation of the LVs of the CLIER, and a reconstruction plot to perform a basic check on the effectiveness of the training.

1. Set your working directory in the directory where the files imported from our GitHub are stored.
2. Import the functions necessary for the process.

> source("aux_functions.R")

***Note:*** The file contains several ready-to-use functions that we created to make the implementation of the protocol faster for new users. This step, in addition to importing the functions that are necessary for the process, also installs (if not yet installed) the R packages used by such functions and loads them.

3. Download the TCGA TPM gene expression dataset.

> dt_tcga <- downloadTcga(save = "tcga.rds")

***Note:*** The function downloads the dataset using the recount3 library, thus providing data processed according to the recount3 pipeline. We will comment on the importance of this specific data processing in the next sections. The argument save is optional, and if desired can be used to store the downloaded dataset in a rds file. In the next steps, several functions have an optional argument save too.

4. Load the previously created single-cell signatures atlas:

atlas <- readRDS("kidney_atlas_matrix.rds") # customize

***Note:*** As mentioned above, in this example we use the kidney-specific atlas constructed to train K-CLIER. Please customize with your own single-cell signatures atlas.

5. Preprocess the TCGA dataset.

> res_prep <- trainPreprocess(dt_tcga, atlas)

[1] "63856 genes and 11348 samples initially."

[1] "63856 genes and 11348 samples after NA removal."

[1] "40714 genes and 11348 samples after conversion from ensembl_gene_id to hgnc_symbol."

[1] "33789 genes and 11348 samples after removal of genes with large number of zeros."

[1] "18157 genes and 11348 samples after filtering genes in no signature."

***Note:*** This step performs the following operations on the TCGA TPM dataset. (1) it restricts the dataset to complete cases by removing any rows with missing values (NA).(2) It converts gene Ensembl IDs to HGNC symbols. Note that this step reduces the dataset's dimensionality as some Ensembl IDs are not associated with any HGNC symbols. Additionally, the correspondence between Ensembl IDs and HGNC symbols is not always one-to-one. If there are multiple matches, only the first match in alphabetical order is used. Next, genes with a high number of zero expressions are removed, specifically those that are zero for more than 95% of the training samples. This threshold can be adjusted using the optional parameter θ. The dataset is then restricted to genes included in at least one single-cell signature from the atlas. Finally, gene expressions are standardized by calculating z-scores, and the mean and standard deviation for each gene are stored.

6. Train the PLIER algorithm.

> clier <- PLIER(dt_tcga, atlas, k=700, trace=FALSE, scale=FALSE, frac=0.5)

***Note:*** The parameter k represents the number of LVs generated by the PLIER algorithm. The value k = 700, has been determined following the guidelines given by Mao et al.,^2^ and depends on the size of the training dataset. The parameter frac, representing sparsity of the decomposition, has been set to 0.5, instead of the default value 0.7. The reduced frac value produces a sparser matrix, which is more suitable for interpretation purposes. No scaling is used (scale = FALSE) since the data have been standardized in the previous step.

7. Add preprocessing information to the trained object.

> clier[["trainStats"]] <- res_prep$dt_stats

***Note:*** This step is necessary since we standardized gene expression in the training dataset. Therefore, we need mean and standard deviation for each gene to transform gene expression to z-scores in any new dataset to which the transformation is applied.

8. Rename LVs in the trained object.

> clier <- renameLvs(clier)

***Note:*** This step is not necessary for the overall functioning of the process, but we prefer renaming the LVs with names LV001, …, LV700. In the original functions, the LV names are built using their index, with appended the name of the gene set to which they are most strongly associated (if any). However, we prefer to remove the second part of the name, as we are interested not only in the most associated signature but also in the other associated ones.

9. Export association information in a user-friendly and readable format.

> dt_info <- reshapeUInfo(clier, save = "associations.xlsx")

> head(dt_info)

 LV name                  Signature

  <char>                   <char>

1:  LV003   Young_T9_Non-Immune_Tumor_Kidney_Renal_cell_carcinoma

2:  LV003   Kuppe_h_CKD_CD10negative_AnnotationLevel3_Uroethlial_Cells

3:  LV003            Bi_h_mRCC_Tumor_TP2

4:  LV003  Kuppe_h_CKD_CD10negative_AnnotationLevel2_Proximal_Tubule

5:  LV003   Kuppe_h_CKD_CD10negative_AnnotationLevel2_Uroethlial_Cells

6:  LV003 Kuppe_h_CKD_CD10negative_AnnotationLevel3_Distal_Convoluted_Tubule

    U   AUC    FDR   p-value

   <num>  <num>   <num>    <num>

1: 0.11643447 0.8244021 3.059837e-33 3.499814e-34

2: 0.10870443 0.8856035 4.496162e-33 5.179402e-34

3: 0.07113319 0.7561515 4.223714e-47 2.622568e-48

4: 0.04430333 0.7639188 5.583722e-30 7.709550e-31

5: 0.03937182 0.9368488 6.125754e-42 4.679395e-43

6: 0.03564866 0.7092066 3.050745e-20 6.704661e-21

***Note:*** This step creates a summary dataset characterizing the association between a given LV and single-cell signatures, providing the strength of the association (U matrix element) along with measures of its significance (AUC, FDR and p-value). For each LV, the signatures to which it is associated are ordered from the most strongly associated to the least strongly associated. Note that only signatures significantly associated (AUC > 0.7, FDR < 0.05) are considered. This file can be used to analyze the interpretable LVs in subsequent analyses.

***Note:*** Two or more different trainings of CLIER, with very small changes in the settings (e.g., a different random seed for the PLIER function, a few added signatures, or a slight modification to one of the hyperparameters), may result in models characterized by a different number of LVs significantly associated with signatures. Additionally, LVs that are labeled in the same way in the two models (i.e., they have the same numeric index) may exhibit different association patterns, or even be significantly associated with signatures in one model and not in the other. If this happens, one might think that the two models are completely different and would lead to different interpretations. However, the two models are most likely not fundamentally different. In fact, it has been shown that the PLIER algorithm is quite robust to such changes, and in such cases, the produced models are essentially equivalent, with the differences mainly amounting to a different indexing of the LVs. If a training produces an LV landscape that seems excessively different from a previous one, given the minimal differences in the training settings, it is possible to check whether these differences are minor or if the models are truly different. This can be done by following the method proposed by Taroni et al.,^8^ which is shown in problem 3 of the troubleshooting section.

10. Conduct a reconstruction check.

> reconstrPlot(clier, Y = res_prep$Y, B = clier$B, save = "train_rec.png")

**Figure 1:**
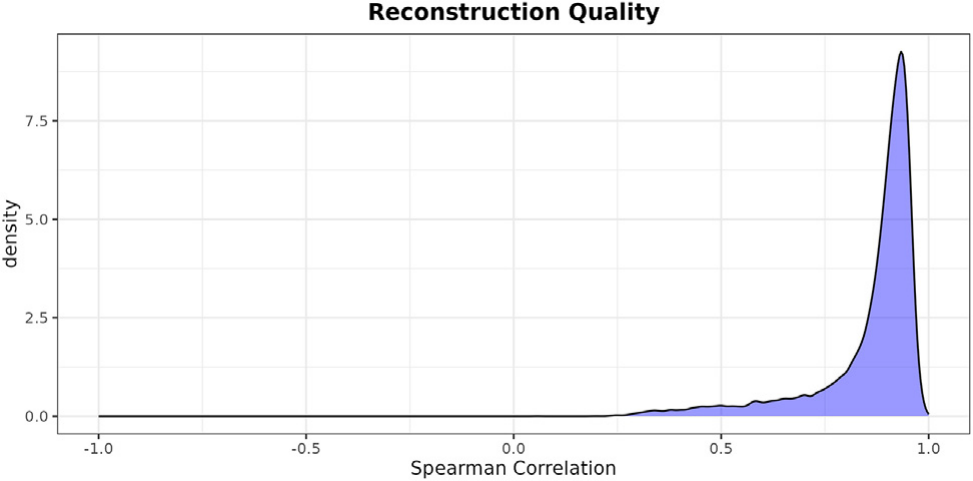
Example of reconstruction plot for the K-CLIER transformation The curve shows effective reconstruction.

***Note:*** Similarly to Taroni et al.,^8^ we verify the effectiveness of the training process by performing an operation analogous to an inversion of the trained transformation. Specifically, we use the matrices created during the training to reconstruct a gene expression dataset, and we measure the Spearman correlation between the true gene expression and the reconstructed gene expression. The plot in Figure 1 shows the distribution of these correlations. A reconstruction producing a peak close to one denotes that the loss of information during the dimensionality reduction was minimal, thus suggesting that the transformation is effective. On the other hand, a wide peak further away from one denotes that the transformation is ineffective and a relevant part of the information was lost.

11. Save the trained transformation.

> saveRDS(clier, "kclier.rds") # customize

***Note:*** As in the example we use the kidney-specific atlas, we call the file kclier.rds. Please customize with your own name.

### Processing new data: Creating a TPM dataset with recount3 pipeline

**Timing: 27 h for 36 samples (approx. 45 min per sample)**

This major step performs a full processing of new data from FASTQ files to TPMs. This entails creating a reference genome, aligning reads, quantifying gene expression in terms of raw counts and extracting TPMs.

***Note:*** As mentioned above, we consider the DKD dataset as an example.

12. Open a terminal, create a conda environment and install STAR aligner 2.7.10a.

$ conda create -n star_aligner

$ conda install bioconda::star=2.7.10a -n star_aligner

13. Retrieve genome reference fasta (GRCh38.p12) and Gencode annotations (v.29).

$ wgethttps://ftp.ebi.ac.uk/pub/databases/gencode/Gencode_human/release_29/GRCh38.primary_assembly.genome.fa.gz

$ wgethttps://ftp.ebi.ac.uk/pub/databases/gencode/Gencode_human/release_29/gencode.v29.annotation.gtf.gz

***Note:*** It is important to specify the version of both references, as they must match those used in the processing of the TCGA operated by the recount3 library.

14. Generate the STAR genome index.

$ conda activate star_aligner

$ GENOME_FASTA=GRCh38.primary_assembly.genome.fa

$ GENOME_GTF=gencode.v29.annotation.gtf

$ STAR_GENOME=/yourstargenomefolder # customize

$ ncpus=32 # specify number of cpus available

$ STAR --runThreadN ${ncpus} --runMode genomeGenerate --genomeDir ${STAR_GENOME} --genomeFastaFiles ${GENOME_FASTA} --sjdbGTFfile ${GENOME_GTF}

***Note:*** This command runs STAR in genome generation mode and stores in the directory specified by STAR_GENOME the STAR genome index file to be used in the next step by the STAR aligner to efficiently map RNA-seq reads to the reference genome.

15. Download .fastq files and align reads to quantify gene expression in the form of raw-counts by running the script align_fastq.sh provided on our GitHub.

#!/bin/bash

# Define the array of URLs

URL_LIST=( "ftp://ftp.sra.ebi.ac.uk/vol1/fastq/SRR106/031/SRR10691631/SRR10691631_1.fastq.gz"

"ftp://ftp.sra.ebi.ac.uk/vol1/fastq/SRR106/031/SRR10691631/SRR10691631_2.fastq.gz")

# Define the fastq files directory

FASTQ_DIR=/fastq_folder

# Create the directory if it doesn't exist

mkdir -p "$FASTQ_DIR"

for URL in "${URL_LIST[@]}"; do

 # Extract filename from URL

 FILE_NAME=$(basename "$URL")

 # Download the fastq file

 curl -L "$URL" -o "$FASTQ_DIR/$FILE_NAME"

 # Check if the download was successful

 if [ $? -eq 0 ]; then

 echo "Download of $FILE_NAME completed successfully."

else

 echo "Error in downloading $FILE_NAME."

fi

done

OUTPUT_DIR=/output_folder

for R1 in ${FASTQ_DIR}/∗_1.fastq.gz

do

  # Derive R2 file by replacing "_1.fastq.gz" with "_2.fastq.gz"

  R2=${R1/_1.fastq.gz/_2.fastq.gz}

  # Extract the sample name (e.g., SRR10691631 from SRR10691631_1.fastq.gz)

  sample_name=$(basename ${R1} _1.fastq.gz)

  # Define the output prefix

 output_prefix="${OUTPUT_DIR}/${sample_name}."

  # Run STAR alignment

  STAR --runThreadN $ncpus \

  --genomeDir ${STAR_GENOME} \

  --outFileNamePrefix ${output_prefix} \

  --readFilesIn ${R1} ${R2} \

  --readFilesCommand zcat \

  --quantMode GeneCounts \

  --twopassMode Basic

done

***Note:*** This script automates the process of downloading FASTQ files and aligning paired-end RNA-seq data using the STAR aligner. As an example, we download the FASTQ files (forward - R1, and reverse - R2) corresponding to the first sample (GSM4217781) of the DKD dataset (GEO: GSE142025). The above code downloads FASTQ data from direct links that can be obtained using, e.g., sra-explorer (https://github.com/ewels/sra-explorer). Alternatively, this data can also be downloaded using the SRA-toolkit (https://github.com/ncbi/sra-tools) with the fastq-dump command and the --split-files flag.

***Note:*** After the download, the script loops through all paired FASTQ files within a specified directory, performs the alignment for each sample, and saves the output to a designated output directory. The script assumes that paired-end FASTQ files are named with _1.fastq.gz and _2.fastq.gz suffixes, respectively. The script is designed to process multiple RNA-seq samples in batch mode and uses multithreading. To use it to download and process FASTQ files you must: (1) update the URL_LIST with the list of direct links obtained from http://sra-explorer.info/; (2) update the FASTQ_DIR to point to the folder containing the FASTQ files; (3) set OUTPUT_DIR to the desired output location for STAR alignment results; (4) ensure that the appropriate STAR genome reference is set in the STAR_GENOME variable.

16. Open R or RStudio, and perform the final processing step, creating a TPM file.

> source("aux_functions.R")

> convertCountsToTpm(folder = "yourstargenomefolder", lengths = “genelength.txt”, save = "DKD_tpm.rds") # customize

***Note:*** The function takes read counts in separate .tab files from a given folder (specified in dir) and the gene lengths file provided on our GitHub as inputs and produces a TPM dataset as an output. The .tab files to be used are selected by providing the directory where they are stored (dir argument) and a common pattern contained in their names (pattern argument). First, it divides the read counts by the length of each gene in kilobases, giving reads per kilobase (RPK). Next, all the RPK values in a sample are summed and this number is divided by 1,000,000 to obtain the 'per million' scaling factor. Finally, the RPK values are divided by the 'per million' scaling factor to obtain TPM. Please customize the input folder name and the output file name.

### Applying the trained transformation to processed new data

**Timing: approx. 3 min**

This major step provides a lower dimensional representation of a new dataset. It takes as inputs a trained CLIER and a new dataset that has been processed following the recount3 pipeline detailed in the previous step. It produces as an output a representation of the dataset in terms of 700 LVs.

***Note:*** In case this step is executed in the same session as CLIER training, the first three operations are not necessary, as those objects are already loaded in R’s global environment.

***Note:*** In the example, we apply K-CLIER to the DKD dataset.

17. Import the functions necessary for the process.

> source("aux_functions.R")

18. Load the previously trained transformation.

> clier <- readRDS("kclier.rds") # customize

***Note:*** Please customize with your own trained CLIER.

19. Load the dataset to be transformed, which has been previously prepared as detailed above.

> dt_new <- readRDS("./DKD_tpm.rds") # customize

***Note:*** Please customize with your own TPM file.

20. Preprocess the new dataset.

***Note:*** The preprocessing of a new dataset differs slightly from that of the training dataset and involves three distinct steps. (1) It restricts the dataset to complete cases by removing any rows with missing values (NA). (2) It converts gene Ensembl IDs to HGNC symbols. (3) It restricts the dataset to only the genes that are common with the training dataset, setting to zero any gene present in the training dataset but not in the new dataset. Finally, it converts the gene expression values to *Z* scores using the means and standard deviations calculated during the training step, which are stored in clier$trainStats.

> Y_new <- testPreprocess(dt_new, clier$trainStats)

[1] "58676 genes and 36 samples initially."

[1] "58676 genes and 36 samples after NA removal."

[1] "40600 genes and 36 samples after conversion from ensembl_gene_id to hgnc_symbol."

[1] "18148 genes and 36 samples after taking only genes common to train."

21. Obtain the LV representation of the new dataset using the trained decomposition.

> B_new <- getB(clier, Y_new, save = "DKD_B.rds") # customize

***Note:*** This step generates the actual lower dimensional representation of the new dataset, which will be used in all the subsequent analyses. Please customize the file name.

22. Build a correlation plot as a check.

> reconstrPlot(clier, Y = Y_new, B = B_new, save = "new_dataset_rec.png")

**Figure 2:**
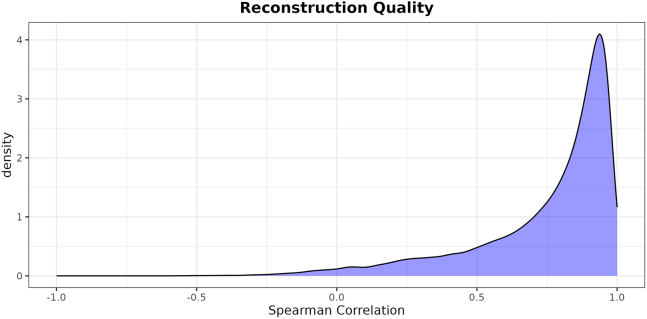
Reconstruction plot for the use of the K-CLIER transformation on the DKD dataset The curve shows effective reconstruction.

***Note:*** Analogously to the training procedure, see step 10; a reconstruction check can also be performed when the transformation is applied to a new dataset (Figure 2). The dimensionality reduction on the new dataset is effective if the reconstruction plot shows a distribution that is not too different from that obtained in the reconstruction check on the training dataset.

***Note:*** If you observe a low correlation in the reconstruction plot, please refer to problem 4 in the troubleshooting section for further guidance.

### Identifying and analyzing relevant latent variables for the study of interest

**Timing: from 30 min to a few days for more complex analyses**

This major step provides an example of how the low-dimensional dataset produced by applying a trained PLIER to new data can be used for further analysis.

***Note:*** In some cases, the transformation may be used with the main aim of reducing dimensionality, thus retaining both the interpretable and non-interpretable LVs. In other cases, the focus may be specifically on the interpretable LVs. In these cases, the analyses may be targeted to identify the LVs most associated with a given outcome, in order for example to extract biological insights, as in the main manuscript associated with this protocol. We provide an example of this second situation.

***Note:*** Relevant variables can be identified either through a univariate approach (e.g., statistical tests) or through a multivariate one (e.g., training a machine learning model and interpreting variables using SHAP). As there are several different ways of doing this, depending on the precise scope of the analysis, we provide an example of the simplest procedure that could be followed, which is conducting Mann-Whitney tests using a binary outcome. In our example, we test the association of the K-CLIER LVs with fibrosis, using the DKD dataset transformed through the K-CLIER.

23. Import the functions necessary for the process (if performed in a new session).

> source("aux_functions.R")

24. Load a dataset with clinical information characterizing the samples.

> dt_clin <- readRDS(“DKD_clin.rds”) # customize

***Note:*** Please customize with your own clinical dataset. In our example, the dataset contains a column “Sample”, with sample IDs, and a Yes/No column “Fibrosis”.

25. Load the LV representation of the new dataset and convert it to a data table for the subsequent analyses.

> B <- readRDS("DKD_B.rds") # customize

> dt_lv <- as.data.table(t(B), keep.rownames = "Sample")

***Note:*** Customize with your own previously obtained LV matrix.

26. Restrict to interpretable LVs.

> dt_info <- readxl(“associations.xlsx”)

> interp_lvs <- unique(dt_info$LV)

> dt_lv <- dt_lv[, c(“Sample”, interp_lvs), with = FALSE]

***Note:*** Since the focus here is on interpretation, we remove the LVs that do not have an interpretation and test only the interpretable ones, thus reducing the impact of corrections due to multiple testing.

27. Conduct Mann-Whitney tests.

> dt_mw <- lvsMannWhitney(dt_lv, dt_clin, on = “Fibrosis”) # customize

> head(dt_mw, 10)

 LV name  p-value Adj. p-value    Regulation

1:  LV002 2.514412e-09 1.961241e-07  Upregulated

2:  LV270 4.310420e-09 1.961241e-07  Downregulated

3:  LV005 1.077605e-08 1.961241e-07  Upregulated

4:  LV596 1.077605e-08 1.961241e-07  Upregulated

5:  LV602 1.077605e-08 1.961241e-07  Upregulated

6:  LV018 2.406651e-08 2.433392e-07  Downregulated

7:  LV056 2.406651e-08 2.433392e-07  Upregulated

8:  LV062 2.406651e-08 2.433392e-07  Upregulated

9:  LV412 2.406651e-08 2.433392e-07  Downregulated

10: LV024 3.484256e-08 3.170673e-07  Upregulated

***Note:*** The name of the binary variable used to define the two populations for the tests is defined through the argument on. The function provides results for Mann-Whitney test conducted using all the available LVs, with a Bonferroni-Hochberg correction for multiple testing. The LVs are sorted in ascending order in the output dataset based on their adjusted p-value. Here we use the variable “Fibrosis”, please customize according to your needs.

**Figure 3:**
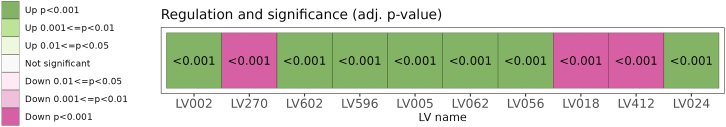
Plot showing the most significant LVs and their regulation *p* values are extracted using the Mann-Whitney test, using Bonferroni-Hochberg correction for multiple testing.

28. Plot the results of the analysis if desired (Figure 3).

> plotSignificantLVs(head(dt_mw,10), save="significant_LVs.png")

29. Inspect the characteristics of the LVs that emerge as significant from the analysis.

***Note:*** This can be done either by reading the Excel file associations.xlsx produced in the previous steps, or by visualizing the structure of LVs as a Sankey diagram, generated using the following piece of code (see next section for a more detailed description of the figure).

> clier <- readRDS("kclier.rds") # customize

> atlas_details <- read_excel("kidney_atlas_info.xlsx", sheet = 2) %>% as.data.table() # customize

> plotSankey(clier, atlas_details, LVs_to_plot=c("LV002", "LV270")) # customize

***Note:*** Plotting the Sankey diagram may require adjustments to parameters controlling graphical aspects such as width, margins, and more. The function in the example above uses default parameters (which is why they are not explicitly shown), but these can be customized as needed. A description of the available parameters is provided in the function code.

## Expected outcomes

This protocol enables learning a context-specific transformation of bulk RNA-seq data. Our approach focuses on selecting signatures of interest, such as those relevant to kidney cells in our example, to specialize the transformation for investigating specific cell types or states and their effects on clinical conditions. After choosing the cell signature atlas, the PLIER method is applied to a large and relevant training set, to obtain the model (here referred to as the CLIER) that will allow transforming the dataset under analysis into a smaller (partially) interpretable space of LVs. “Interpretable” in this context indicates that some of the LVs are associated with at least one single-cell signature in the provided atlas. By accounting for correlations between genes, and being driven by the prior knowledge represented by the atlas, these LVs can better capture biological mechanisms than individual genes alone and serve as new features that can be correlated with clinical data of interest to construct biologically interpretable models.

In the example of the section “identifying and analyzing relevant latent variables for the study of interest,” we identify the LVs significantly correlated with an outcome of interest (fibrosis) through hypothesis testing; more advanced analytical tools and models including machine learning, could be used to analyze such associations. The CLIER approach provides the means to interpret the LVs emerging from the correlation analysis. The association of a given LV to signatures can be easily retrieved in the Excel file (named associations.xlsx in the example above) saved at the end of the training (major) step. The file includes the list of pairs (signatures, LV) associated with AUC bigger than 0.7 with the corresponding U matrix element which quantifies the strength of the association: a big U-value for the pair (Signature A, LV1) implies that a change in the value of LV1 has a big impact in the expression of signature A genes.

**Figure 4:**
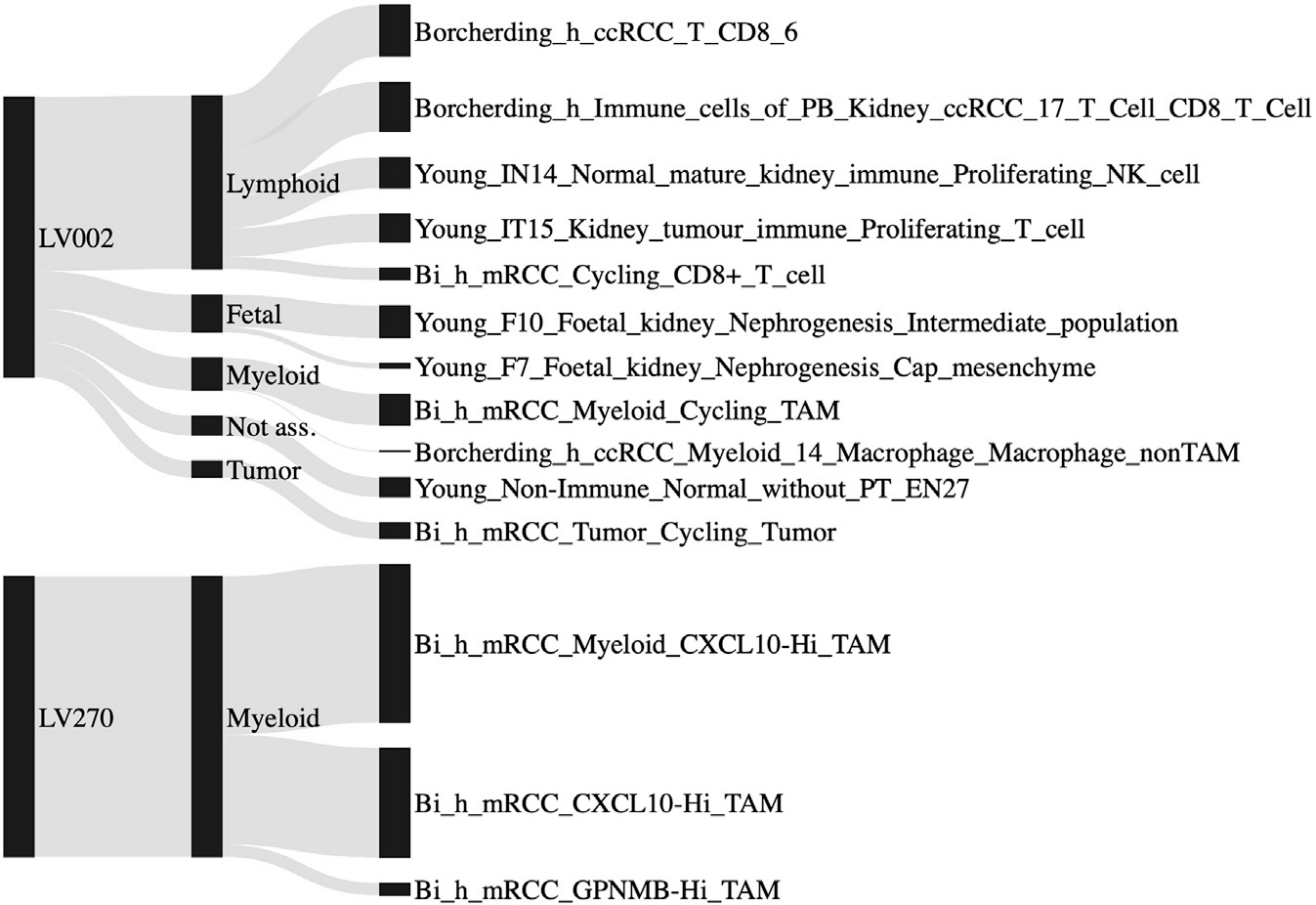
Example of latent variables from K-CLIER and their association with single-cell signatures Note how LV002 is mainly associated with Lymphoid cell type, whereas LV270 is only associated with Myeloid cell type.

As an illustration of how this information can be effectively visualized to characterize LVs, we show in Figure 4 the Sankey diagrams built to interpret two LVs of the K-CLIER (in particular, we show two variables that emerge as significant in the analysis at step 27). Considering that the kidney atlas contains signatures linked to several cell types and states, we found it beneficial to group them into groups, each associated with a distinct cell type or state. For each LV, the second layer of the Sankey diagram assigns to each group the sum of the U-values of the signatures in the group while the third layer shows the U-value of each specific signature (here we set the height of the first layer to have the same length for both LVs).

## Limitations

The main limitation of the proposed method is that the data to which CLIER is applied need to be processed in the exact same way as the training TCGA dataset (i.e., following the recount3 pipeline). Therefore, applying the transformation to a dataset from a previous publication that is available on GEO in TPM may not be feasible and may require instead a full reprocessing starting from the FASTQ files, which can be time-consuming.

Moreover, even when the training and test datasets have been processed in the same manner, and even though the focus on biologically meaningful latent variables is expected to mitigate negative effects of technical noise, it cannot be ruled out that the presence of batch effects in the datasets used may impact the validity of the transformation.

## Troubleshooting

### Problem 1

The published single cell signatures do not describe the biology of interest for the given study (extracting single-cell signatures from literature, step 2).

### Potential solution

To define LVs that are relevant to the problem of interest, the single cell signatures used as inputs to train the PLIER algorithm should contain similar cell types and the same condition of interest. Otherwise, it is necessary to perform experiments to extract single cell signatures that are relevant to the problem of interest. The challenge is to design relevant experiments that are as close as possible to the problem under investigation. Particular attention must be paid to the species involved, which should ideally be identical to the bulk RNA-seq dataset of interest.^9^ Similarly, the main interest of snRNA-seq is to detect very fine sub-population changes in relation to the disease model. The closer the reference signature is to the disease model studied by bulk RNA-seq, the greater the chance of finding a relevant LV. Finally, snRNA-seq datasets should be integrated and annotated very carefully, following gold standard pipelines.^10^

### Problem 2

The user is not in possession of high-memory servers (materials and equipment).

### Potential solution

While high-memory servers are ideal, STAR can be adapted to local stations with optimizations to parameters that reduce memory and runtime.

According to the STAR manual, STAR requires around 30 GB of RAM for aligning reads to large genomes such as the human genome. The memory usage can be adjusted by optimizing specific parameters, including --genomeChrBinNbitsand --genomeSAindexNbases, to reduce RAM consumption (see the STARmanual for details).

We tested our protocol on a local station with the 8 cores, 64 GB RAM, SSD storage. Under these conditions, the following runtimes were observed:

• Genome Indexing (human genome): about 4–6 h.
• Read Alignment (30 M paired-end reads): about 1 h per sample.

Users with more constrained computational resources can consider the following:

• Reduce the number of threads (--runThreadN) based on available CPUs.
• Use the --limitBAMsortRAM parameter to limit memory usage during BAM file sorting.
• Utilize --genomeLoad LoadAndKeep to preload genome indices into shared memory when processing multiple samples in one run, which reduces memory overhead by reusing the genome index.

### Problem 3

A new training of CLIER, with slightly different training settings, determines a LVs landscape that appears significantly different from the previous one (training PLIER algorithm, step 9).

### Potential solution

**Figure 5:**
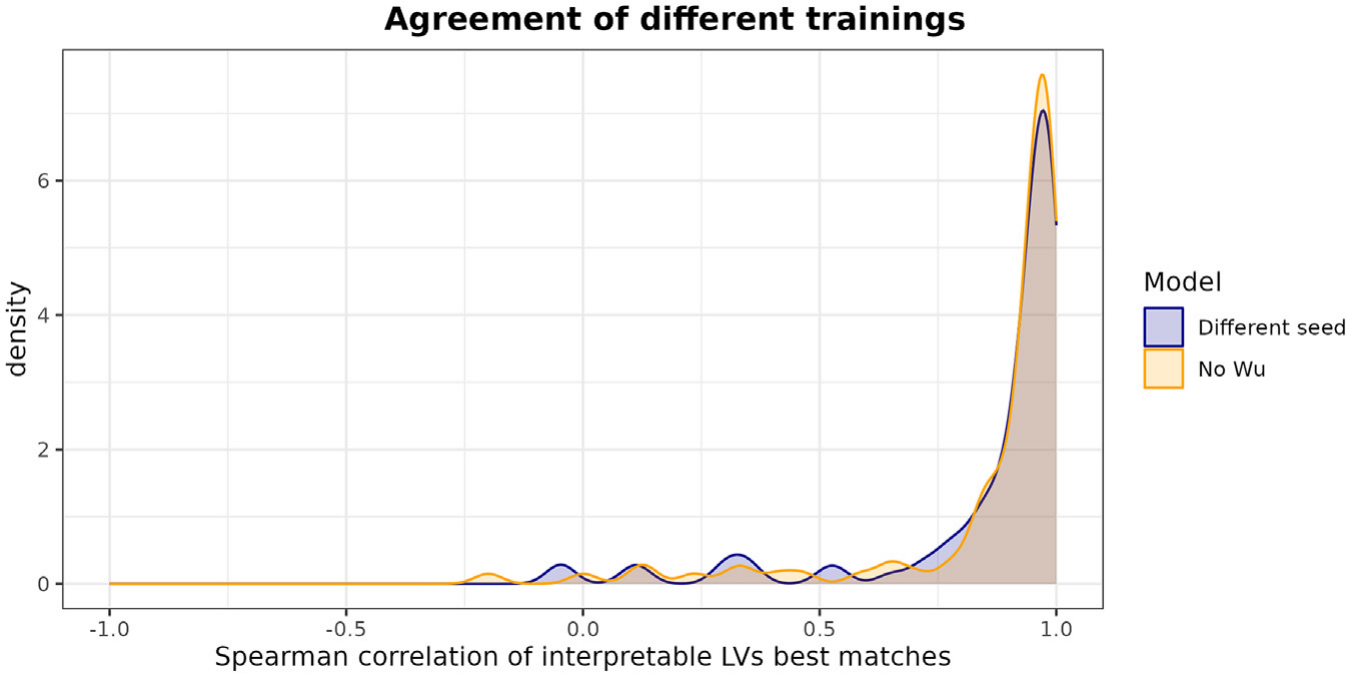
Agreement with reference of two different models For the first model, a different random seed was used. For the second model, the 14 signatures extracted from Wu et al.^11^ were removed. Both models show good agreement with the reference model, despite exhibiting some differences in the LV landscape, including a difference in the number of LVs significantly associated with signatures.

It is possible to check whether the differences are only limited or if the models are truly different by following a procedure described in Taroni et al.^8^ In this case, we aim to establish equivalence for the interpretable LVs, while the non-interpretable ones, which are not constrained by the signatures, are expected to show greater variation. Let us consider a reference model and a test model. We examine the interpretable columns of the Z matrices of the two models and identify which LVs in the test matrix are best correlated with those in the reference matrix. Note that the correspondence may not be one-to-one, as the two models may even possess different numbers of interpretable LVs. For the best matches, we calculate the correlations using the corresponding rows of the reference and test B matrices. If the two models agree, this will produce a density plot peaked towards one, as shown, for example, in Figure 5. If this is the case, the difference between the models is not an actual problem and does not require a solution. If this is not the case, the user should investigate the reason for the disagreement, including reviewing the code for potential errors. It is worth mentioning that, on the other hand, poor correlation patterns within the sets of non-interpretable LVs would not necessarily imply non-equivalence between the two models. In fact, since those variables are not aligned with prior knowledge, a strongly consistent correlation pattern is not expected.

**Figure 6:**
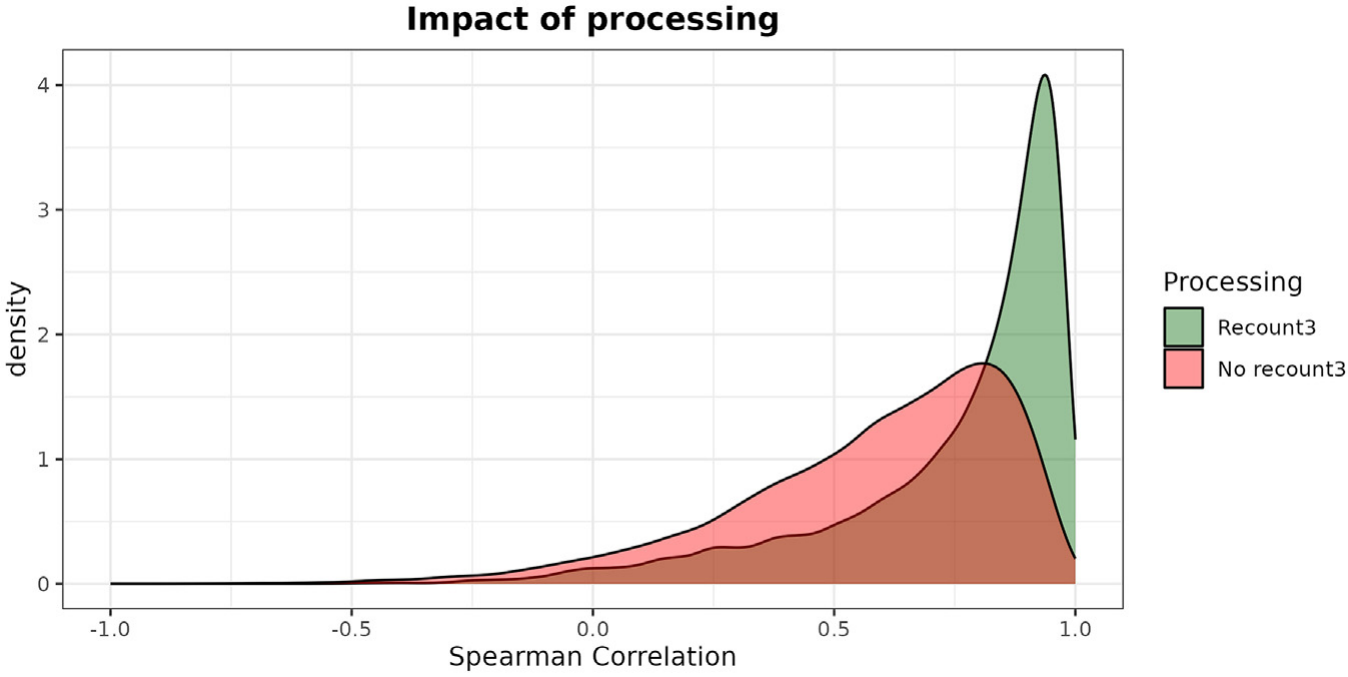
Comparison of reconstruction plots when K-CLIER is applied to two different versions of the DKD dataset One version is processed with the recount3 pipeline (green), the other with a different pipeline (red).

### Problem 4

The reconstruction plot does not yield a satisfactory result (applying the trained transformation to processed new data, step 22).

### Potential solution

Poor reconstruction quality after applying a trained PLIER to a new dataset may stem from processing issues. It is crucial that the dataset to which the transformation is applied undergoes identical processing as the training dataset, including using the same software versions. Therefore, a closer examination of the processing steps (including versions) could be beneficial if the reconstruction quality is unsatisfactory. As we download TCGA processed with recount3, we developed a pipeline that adheres to this approach. To illustrate the importance of identical processing, we provide a reconstruction plot (Figure 6) comparing the application of CLIER to two differently processed versions of the DKD dataset (one processed with our pipeline, and labeled as recount3, and one downloaded directly from the GEO website and already processed in a different way).

## Resource availability

### Lead contact

Further information and requests for resources should be directed to and will be fulfilled by the lead contact, Anna Rinaldi anna.rinaldi@eoc.ch.

### Technical contact

Questions about the technical specifics of performing the protocol should be directed to and will be fulfilled by the technical contact, Daniele Malpetti daniele.malpetti@idsia.ch.

### Materials availability

This study did not generate new unique reagents.

### Data and code availability

The code generated during this study is available at https://github.com/IDSIA/CLIER (https://doi.org/10.5281/zenodo.14272914). At the same link example data are also available. The K-CLIER model, together with other files produced as intermediate steps during the execution of the pipeline, can be downloaded at the following link: https://drive.switch.ch/index.php/s/OpvMh1vGRgRmKKf.

## Acknowledgments

The project was funded by the Swiss Kidney Foundation and by a research grant of the Ente Ospedaliero Cantonale in collaboration with the Istituto Dalle Molle di Studi sull'Intelligenza Artificiale.

The research of D.L. is supported by two young research grants from the Geneva University Hospital (PRD 5-2020-I and PRD 4-2021-II) and by a grant from the Ernst and Lucie Schmidheiny Foundation.

Work in P.C.’s laboratory is supported by a grant from the Switzerland National Foundation (Sinergia, CRSII5_202302) and by the Balli and the Gianella foundations.

We thank the staff of the intensive care unit of the university hospital of Geneva for supporting the project during the challenging time of the COVID-19 pandemic. We thank the team of the genomic facility of the Institute of Oncology Research, Bellinzona.

The graphical abstract makes use of the following icons from thenounproject.com CC BY 3.0: “Paper” by creative outlet, “Arrow” by Alice Design, “Machine Learning” by Mohamed Mb, “Lens” by Kotti, “rna” by huijae Jang, “Machine Learning” by Diheksa26, and “histogram” by New River.

## Author contributions

Conceptualization, F.M., A.R., D.M., P.C., and L.A.; formal analysis, D.M. and F.M.; data curation, D.M., A.R., and M.B.; writing, D.M., F.M., M.B., A.R., D.L., and L.A.; supervision, P.C. and L.A.; funding acquisition, L.R. and P.C.

## Declaration of interests

The authors declare no competing interests.
